# Steroid utility, immunotherapy, and brain tumor management: an update on conflicting therapies

**DOI:** 10.37349/etat.2022.00106

**Published:** 2022-10-31

**Authors:** Matthew Goldman, Brandon Lucke-Wold, Meleine Martinez-Sosa, Jason Katz, Yusuf Mehkri, Jeff Valisno, Stephan Quintin

**Affiliations:** 1 UFCOM, Gainesville, FL 32608, USA; 2 Department of Neurosurgery, University of Florida, Gainesville, FL 32608, USA; 3 John Hopkins A1l Children’s Hospital, St. Petersburg, FL 33701, USA; Istituto Nazionale Tumori “Fondazione Pascale” Via Mariano Semmola, Italy

**Keywords:** Steroids, tumor, management, algorithm

## Abstract

Steroid use is a widely accepted practice for both the treatment and prevention of tumor-induced edema, but there are many unknowns regarding their current clinical utility with modern anti-tumor therapies. This decreases edema and relieves the symptomatic mass effect. There are clearly understood benefits and commonly accepted complications of methylprednisolone (MP) use, but the topic is recently controversial. With immunotherapy advancing, a robust immune response is crucial for full therapeutic efficacy. The immunosuppression of MP may interfere with future and current therapeutics relying on the integrity of the patient’s immune system. This further emphasizes the need for alternative agents to effectively treat tumor-induced cerebral edema. This review highlights the current clinical utility of steroids to treat brain tumor-related edema and the underlying pathophysiology. It also reviews details regarding different steroid formulations and dosing. Research available regarding concurrent steroid use with immunotherapy is detailed next, followed by alternatives to steroids and barriers to their adoption. Finally, this paper discusses pre-clinical findings and emerging treatments aimed to augment or replace steroid use.

## Introduction

A large percentage of patients with both primary and metastatic brain tumors experience symptomatic edema. The volume of mass effect secondary to tumor-induced vasogenic edema is often greater than that produced by the tumor itself [1]. Historically glucocorticoids have been used to provide symptomatic relief since the late 1950s [2]. Methylprednisolone (MP) administration has been proven to increase tight junction expression and support the integrity of the blood-brain barrier (BBB) [3]. The administration of steroids can improve headaches and neurologic deficits by reducing edema and is a powerful tool for neurosurgeons that should be used with caution.

Dexamethasone was the first drug brought to the Federal Drug Administration (FDA) for approval by neurosurgeon Joseph Galichich in 1958 to treat brain tumor-related edema [4]. Extensive advancements in tumor management have occurred since and new questions have emerged. As the evolution of immunotherapy continues to show promise in the treatment of brain tumors [5] the role of steroids is now in flux. If clinicians intend on administering concurrent therapy, important questions on drug interaction and scheduling must be addressed or steroid alternatives developed.

## Current clinical utility

Guidelines released by the National Comprehensive Cancer Network (NCCN) clearly state that steroids should be used sparingly and only when necessary. Recommendations suggest use at the lowest effective dose for the shortest possible time [6]. In addition to symptomatic edema relief, steroids provide post-operative nausea, pain, and appetite benefits [7]. Although benefits and side effects are well established, the indications for initiating steroids and dosing paradigms have changed in recent years. Specific considerations for utility within intracranial tumor subtypes are detailed below. Details regarding steroid selection and treatment algorithms will be discussed later in this review.

A recent paper surveying 175 neurosurgeons across 55 countries provided insights into worldwide perioperative steroid use in clinical practice. The majority (88%) chose to prescribe steroids in the perioperative period, but clinical reasoning differed. Interestingly 45% of surgeons report treating all patients with steroids regardless of symptoms. Of the remaining respondents, 44% reported treating symptomatic patients and 11% reported other reasons for treatment [8].

Although tapering as soon as possible prevents detrimental side effects of steroids, it precipitates diagnostic and clinical challenges for the physician. Tapering from steroids, in addition to radiotherapy (RT) and certain immunotherapies, can increase the permeability of the BBB increasing contrast enhancement and edema [9].

### Glioblastoma multiforme

In cases of recurrent high-grade gliomas, carmustine wafer therapy is being used to bypass the BBB and directly localize chemotherapeutics minimizing systemic side effects [10]. Although effectively improving overall survival, wafers have a high complication rate (42.7%) and are expensive [11]. Following wafer placement steroids, in addition to watertight dural closure, are essential for preventing perioperative adverse events [12].

**Figure 1.**
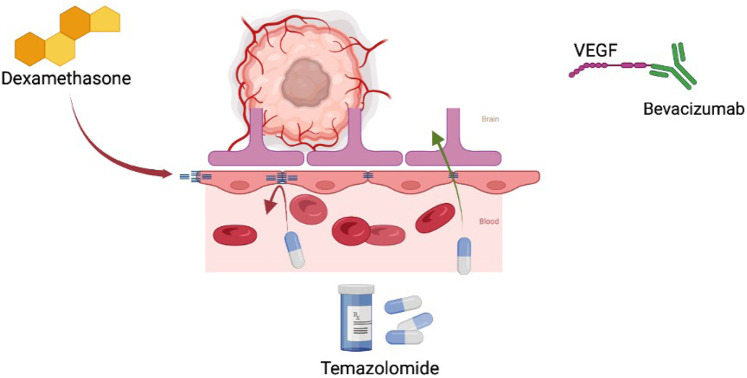
Immunosuppresent effects on the tumor microenvironment. This figure illustrates how dexamethasone upregulates tight junctions of the BBB which may alter pharmacokinetics. It also shows hyperglycemia induced by steroids fueling oncologic anaerobic metabolism. VEGF: vascular endothelial growth factor

A meta-analysis of twenty-two publications including 8,752 patients was published in 2020 by Petrelli et al. [13] investigating the effects steroids have on the outcome when associated with the treatment of glioblastoma multiforme (GBM). This patient cohort was treated with surgery and concomitant temozolomide-based chemoradiotherapy (CTRT). Results of their analysis showed the use of steroids was associated with reduced survival and reduced progression-free survival. Hyperglycemia is a common side effect of steroid use that may help explain worsened outcomes. Another meta-analysis performed by Lu et al. [14] aimed to quantify the effect of hyperglycemia during the management of GBM on overall survival. Their analysis included six retrospective observational studies including 1,481 patients. Results demonstrated that hyperglycemia showed a trend towards worse prognosis consistently across all included studies. One mechanism proposed includes a direct link to tumor progression secondary to increased metabolic substrates for anaerobic glycolysis, hyperglycemia-induced hyperinsulinemia stimulating tumor growth, and hyperglycemic dysregulation of innate protective mechanism in the tumor microenvironment (see Figure 1). The results of these studies further reinforce principles established by NCCN guidelines, that steroid use should be limited whenever possible [6].

### Metastasis

Brain metastases are the most diagnosed malignant intracranial tumor, most commonly spreading from primary malignancies in the breast, lung, kidney, and skin (melanoma) [15]. Steroids are known to have a profound impact on symptomatic relief from tumor-related edema. Up to 75% showed marked neurological improvement within 24–72 h after initiating dexamethasone treatment [2]. The management of metastatic brain tumors has evolved over time and a multitude of treatment options have emerged over the past twenty years. Traditionally whole brain radiation therapy (WBRT) has been the standard treatment for multifocal brain metastases, but cognitive dysfunction limited clinical utility. Recently innovations in various stereotactic irradiation (STI) techniques including linear accelerators, Gamma Knife, and CyberKnife have shown promise in treating metastases with minimal radiation delivered to surrounding tissue [16]. Steroids are not only useful to manage vasogenic edema but additionally headaches secondary to radiation treatment [17].

Stereotactic radiosurgery (SRS) now plays an important role in handling inoperable metastatic brain tumors. One of the side effects of RT is radiation-mediated damage and steroids are of the few therapeutic options to address this. A recent meta-analysis done by Lamba et al. [18] found symptomatic radiation necrosis requiring steroid or other intervention was required in 27% of patients receiving SRS. The topic of concurrent immune checkpoint inhibitors (ICIs) is especially relevant for the treatment of brain metastasis considering the ongoing utilization of these therapies. The current understanding and management of steroids given concurrently with these therapies will be discussed later in the paper.

### Primary central nervous system lymphomay

Primary central nervous system lymphoma (PCNSL) requires specific caution with steroid administration. Steroids provide rapid symptomatic relief and radiographic improvement but may lead to nondiagnostic biopsies with altered histopathology. If imaging raises suspicion of central nervous system (CNS) lymphoma, steroids should be held until a biopsy has been performed. If steroids have already been given, a biopsy may have to be repeated after steroid cessation [6]. PCNSL is a potentially curable disease and effective management depends largely on establishing a diagnosis, which is often difficult involving a broad differential diagnosis. To avoid delayed treatment, alternative agents such as hyperosmolar therapy can be used to relieve symptomatic edema before a diagnostic biopsy [19].

## Pathophysiology

### Rationale and mechanisms

Cerebral edema comes in three main forms: vasogenic, cytotoxic, and interstitial [20]. Vasogenic edema reflects the extravasation of intravascular fluid to the extracellular space in the setting of an impaired BBB. This can be seen in malignancy, trauma, or infection. This barrier impairment is a consequence of many factors including endothelial disruptions, paucity of normal astrocytes, and tumor-secreted factors like VEGF. Glucocorticoids are helpful for brain tumor edema via the reduction in tumor capillaries and strengthening of the BBB [21, 22].

Steroids are non-polar substances and easily traverse into the cytoplasm when unbound [23]. Subsequent binding to cytoplasmic receptors allows this couple to migrate to the nucleus where ultimately protein expression is affected. Pro-inflammatory cytokines are decreased while further regulatory control is achieved via transcription factor modulation [i.e. nuclear factor kappa B (NF-kB)]. Specific targets may include increased angiopoietin 1 and decreased VEGF [22]. Furthermore, the BBB is reinforced via the corticosteroid-induced increase in tight junction components like occluding (see Figure 1). Glucocorticoids may also treat patients with brain tumors via direct inhibition of cellular proliferation and augmentation of apoptosis [23]. Multiple studies have shown that these seemingly microscopic mechanisms result in positive gross changes radiographically [24, 25].

Interestingly, acute mountain sickness (hypobaric, hypoxic conditions) has been shown to produce intraparenchymal similarities to those with brain tumors (i.e. vasogenic edema, increased intracranial pressure). Dexamethasone is effective in treating acute mountain sickness, likely due to its effects on bio factors like interleukin 1 (IL-1) receptor agonist (IL-1RA), heat shock protein 70 (HSP-70), and adrenomedullin. Dexamethasone-mediated IL-1 suppression has been shown to be effective in mouse models of glioblastoma-associated cerebral edema [26]. Interestingly, dexamethasone administration during radiation for GBM cohorts was associated with decreased survival [27, 28].

### Standard of practice

From a clinical practice standpoint, steroid use in brain tumor management is highly controversial and varies not only across tumor types but also by institution and physician. This is largely due to the complications that have been shown to be associated with preoperative steroid use in neurosurgical procedures. One study focused on patients undergoing decompressive craniotomy and spine surgery, using data from over 25,000 neurosurgical procedures recorded in a national database, and found significant associations between preoperative steroid use and postoperative infection [29]. Although these findings have not been replicated in patients undergoing craniotomy for tumor resection, an increased risk of readmission has been noted [30]. Others are concerned about the side effects associated with steroid use, such as hyperglycemia. One study by Hempen et al. [30] noted serum glucose levels over 100 mg/dL in about half of all patients with brain metastases and three-quarters of all patients with primary brain tumors in their study who were on dexamethasone treatment. This raises concern due to the poor outcomes that have been associated with hyperglycemia and outcomes in brain tumor patients. Liu et al. [31] conducted a recent meta-analysis on 2,168 patients and found that hyperglycemia was significantly associated with decreased overall survival, and this was irrespective of the cause of hyperglycemia.

Historically, between 70–100% of patients with brain metastases have received steroid treatment during adjuvant RT, and between 10–50% after completion of RT [32–37]. Additionally, dexamethasone appears to have been the steroid of choice for these patients, with a starting daily dose of around 16 mg, but highly dependent on the patient clinical status [31, 35, 38]. Currently, clinical practice guidelines for patients with brain metastases recommend starting these patients on 4–8 mg/day of dexamethasone in the case of mild symptoms related to mass effect and reserving the higher dose of 16 mg/day for patients with severe symptoms [1]. These recommendations were primarily derived from evidence showing a lack of benefit in higher doses for patients with mild symptoms and side effects being dose-dependent. They also recommend tapering these patients off of steroids over a minimum 2-week period to avoid symptom recurrence.

Additional considerations should be taken in patients with GBM. Currently, the golden standard for GBM treatment is maximal surgical resection followed by adjuvant RT and/or temozolomide (TMZ). The current literature has shown poor survival and disease progression in patients dependent on steroid therapy postoperatively and/or during adjuvant RT/TMZ [39–41]. Interestingly, patients on dexamethasone while concurrently being treated with bevacizumab and RT/TMZ have demonstrated no change in overall or progression-free survival [42]. This suggests that steroid therapy may be beneficial in GBM patients experiencing neurologic or global symptoms, especially when administered concurrently with bevacizumab (see Figure 1).

Regarding pediatric brain tumor management, provider perspectives and treatment practices regarding steroid utility have recently been explored [43]. No standard guidelines exist for steroid management in newly diagnosed pediatric brain tumors, contrary to adults where prescribing practices have been suggested. Based on a national survey distributed to pediatric providers involved in the management of newly diagnosed brain tumors, almost all providers agreed that they would start steroids for vasogenic edema and obstructive hydrocephalus, would use dexamethasone, and were more likely to start steroids if they believed the patient would improve within 24 h. In an older pediatric patient, most providers agreed upon a loading dose of 10 mg and a maintenance dose of 4 mg/6 h. They also agreed that they would initiate weaning for a stable patient after 1–3 days with a weaning duration of 5–7 days.

Taking all of this into account, the current standard practice favors dexamethasone therapy for acute management of patients with brain tumors using the lowest dose necessary to improve quality of life, while minimizing the risk of treatment-related complications and impact on survival outcomes. Additionally, the literature does not support steroid treatment in asymptomatic patients. As mentioned in the most recent NCCN guidelines from 2021, steroid therapy should not be avoided when necessary, but every attempt should be made to downward titrate the dose being administered [6]. Additionally, there should be careful monitoring for complications, especially in high-risk patients, such as those on anticoagulation.

## Initiating and maintaining treatment

### Patient selection

American Society of Clinical Oncology states that for patients with asymptomatic brain metastasis without mass effect, there is insufficient evidence to recommend or advise against steroid treatment [44]. This holds true even if imaging reflects peritumoral edema present [23, 45]. For patients with metastatic brain tumors who are symptomatic (i.e. due to increased intracranial pressure, edema, etc.), 4–8 mg/day of dexamethasone should be considered [46]. Higher doses (16 mg/day) may be considered for those with moderate/severe symptoms [44, 46] knowing that side effects may be increased. Tapering should begin as soon as clinically possible (for those with greater than 2–3 weeks of administration) via an individualized approach [47, 48]. Other important considerations are to use the minimum effective dose and avoid night-time doses for minimized toxicity.

Dexamethasone is by far the most widely used and recommend agent (see Table 1). Nevertheless, a study of 48 patients with radiographic brain tumor edema compared MP to dexamethasone and showed comparable rates of clinical neurological symptom improvement [33]. Steroid prescription necessitates disclosure of potential side effects including *pneumocystis carinii* pneumonia, osteoporosis, psychosis, mood disturbance, Cushing’s, cognitive dysfunction, metabolic syndrome, immunosuppression, thrombosis, and myopathy [23, 49]. Additional well-known consequences include fluid retention, visual disturbances, and cerebral atrophy [45]. For all these reasons, it is advisable to use only if indications are met, and only the minimal dose necessary with appropriate close follow-up/titration. Encouragement of exercise is recommended to potentially reduce the risk of steroid myopathy, and some suggest gastric ulcer prophylaxis with H2 blockers to reduce the risk of hemorrhage.

**Table 1.** Most common corticosteroids utilized for tumor edema and associated dosing, pros, and cons

**Name**	**Dosing**	**Pros**	**Cons**
Dexamethasone	- Loading: 10–20 mg intravenous (IV) up to 100 mg/day [23] - Maintenance: 4–8 mg/day (mild symptoms) to 16 mg/day (moderate to severely symptomatic); up to 24 mg/day [23, 44]	- Long half-life (3–4 h with biologic half-life 34–54 h) [45] - Low/absent mineralocorticoid activity, less psychosis, improvement in clinical condition pre-operatively, lower infection risk [23]	- Increased risk of myopathy [23] - May alter the appearance of post-contrast imaging [45] - Decreased survival among GBM patients undergoing radiation [45] - Decreased half-life in the setting of co-administration of phenytoin [47]
Prednisone	- 5–60 mg/day [49]	- Lower risk of myopathy [23] - Allows smaller dose taper for those with adrenal insufficiency history - Less mineralocorticoid potency that hydrocortisone [49]	- Less efficacious for brain edema [23]
Hydrocortisone	- 20–30 mg/day [49]	- Lower risk of myopathy [23, 49]	- Increased mineralocorticoid potency compared to dexamethasone [49] - Shorter biological half-life (8–12 h)

## ICIs

ICIs leverage the patient’s own immune system to fight off tumor cells via the administration of antibodies targeting cytotoxic T-lymphocyte antigen-4 (CTLA-4), programmed cell death protein 1 (PD-1), and PD-1 ligand (PD-L1). These antibodies prevent tumor immune tolerance by blocking ligand-receptor interactions and subsequent effector signaling that ultimately inhibit anti-tumor immune responses [50]. The use of ICIs has revolutionized oncology and induced substantial leaps in the treatment of a wide range of tumors including melanoma and non-small cell lung cancer (NSCLC) [51], both capable of metastasis to the brain. Several studies assessing ICI utility in brain tumors were compiled in a large systematic review and demonstrated a moderate response to ICI in melanoma and NSCLC brain metastases with some clinical effects in glioblastoma [51]. However, whether this moderate response is blunted by steroid use to manage symptomatic tumor-associated edema is still under question [52–56].

Fundamentally, the immunological effects of ICIs and corticosteroids are antagonistic: ICIs boost immune response, whereas corticosteroids are immunosuppressive. There is preliminary evidence that concurrent use is detrimental to the anti-tumor benefits of ICIs reflected in shorter overall survival and systemic progression-free survival associated with corticosteroid use in patients with brain metastases receiving ICI [57]. Therefore, in the setting of symptomatic cerebral edema and ICI use, corticosteroids should be administered judiciously at the minimum dose and period possible [58]. Improvement is typically observed within hours of administration peaking after 24–72 h. Initiate taper when symptoms begin to improve and determine a taper schedule on a patient-by-patient basis [59]. The potentially antagonistic effect of corticosteroids on ICI utility highlights the importance of discovering alternative strategies to manage cerebral edema, especially with concurrent ICI use. Further studies evaluating the timing and dosing of corticosteroid administration in relation to ICI timeline may also reveal time points after the establishment of initial immunity when corticosteroids are less likely to diminish the anti-tumor benefits of ICIs.

Steroid use prior to or after ICI initiation is also important to consider. All-cause mortality was higher in melanoma patients exposed to steroids up to 3 months before ICI initiation compared with non-exposed patients (126% if ICI was initiated ≤ 1 month after steroid exposure, 51% if 1–3 months after exposure) [60]. In a separate study, baseline corticosteroid use initiated ≤ 14 days before or ≤ 30 days after ICI initiation was associated with shorter median overall survival in advanced NSCLC, melanoma, and urothelial cancer [61]. These multi-system results emphasize the importance of considering the lingering effects of corticosteroids on ICI anti-tumor mechanisms even several months before the initiation of ICIs, thereby corticosteroid use within the past year should be considered when gathering the patient’s history. Given the importance of corticosteroids in the management of cerebral edema, a focused study evaluating the effects of corticosteroids with respect to time prior to or after initiation of ICIs could delineate a potential time-dependent association between corticosteroid use and ICI efficacy. Further studies may also reveal time points after the establishment of initial immunity when corticosteroids are less likely to diminish the anti-tumor benefits of ICIs. These studies are crucial to the generation of specific clinical guidelines for the use of steroids on patients in immunotherapy and may improve outcomes for brain tumor patients using ICIs.

## Alternative agents

While steroids have been shown to have broad utility in the context of brain tumor management, they also have broad neurologic and systemic side effects [23, 62]. These side effects are dose-dependent and may regress with cessation of steroid use but often include long-term side effects such as osteoporosis and cataract formation [23, 63, 64]. Side effects limit the use of steroids in managing cerebral edema and inflammation associated with brain malignancies, surgical resection, and RT. Treatments that can be used alternatively or in conjunction with steroids in this context are currently being investigated. The following section will discuss these emerging treatments, their mechanisms, and barriers to their use.

### Bevacizumab

Among current FDA-approved drugs, bevacizumab has seen use as a primary treatment for glioblastoma and for reducing cerebral edema and radiation necrosis [65, 66]. Bevacizumab is a monoclonal antibody against VEGF which is highly expressed in high-grade gliomas [67]. VEGF expression has been shown to promote endothelial cell proliferation and migration leading to disorganized angiogenesis [68]. VEGF expression and blood vessel density are strongly associated with overall outcomes in these cancers [69]. Blocking of VEGF signaling contributes to the side effects of bevacizumab and anti-VEGF treatment which commonly bleeding, prolonged wound healing, and hypertension, but are generally mild [70]. The benefits of bevacizumab are thought to be due to the re-normalization of the cerebrovascular endothelium and subsequently the BBB following treatment [71]. However, investigations into the treatment of edema or attenuation of radiation necrosis with bevacizumab are limited. An early case report documenting the use of this drug for edema, showed, after 24 h of treatment, a reduction in symptomatic cerebral edema in 50 out of 59 patients and a significant edema volume reduction in 55 of the 59 patients [72]. These findings are supported by a more recent retrospective study, which showed that refectory edema in patients with metastatic brain tumors was relieved in 72 of 83 patients treated [73]. Bevacizumab has also been shown to improve neurocognitive outcomes in patients with radiation-induced brain necrosis (RBN) when compared to patients treated with corticosteroids [74–76]. Improved outcomes are also seen in RBN that is refractory to steroids [77]. Despite relative improvement in outcomes, radiological improvement in RBN shown in these studies was not significant compared to steroid treatment alone [74]. A recent systematic review and meta-analysis showed that bevacizumab treatment allowed for the cessation or reduction of steroid use in patients with RMN [75]. Bevacizumab is currently being studied as a chemotherapeutic agent adjunct to other therapeutics in the context of malignant gliomas, but studies thus far have not proven its efficacy in terms of clinically significant improvements in outcomes when used as a direct treatment [67]. While its ability to reduce cerebral edema and improve outcomes secondary to RMN is promising, these benefits over steroids must be clinically significant to justify the additional cost of bevacizumab. While steroid medications are relatively inexpensive, bevacizumab has been shown to increase the cost of cancer treatment by as much as > 200% when used as a primary cancer treatment [70, 78]. Future studies aiming to optimize treatment algorithms to include both bevacizumab and steroids may reduce costs, and side effects, and improve the feasibility of their use in treating secondary complications of brain cancers and cancer treatment.

### Human corticotropin-releasing factor

Preliminary research also suggests that future treatment algorithms may benefit from the addition of corticotropin-releasing factor (CRF) [79–81]. Corticorelin acetate, a synthetic corticotropin-releasing agent has since been shown to reduce the dexamethasone dose needed to manage edema in larger randomized control trials [80]. In addition to increasing endogenous production for corticosteroids, human CRF (hCRF) analogs may have direct anti-tumor activity as shown in preclinical studies [79, 82, 83]. The side effects of hCRF are mild, primarily consisting of hypotension and flushing at high doses [80]. hCRF has been shown to reduce dexamethasone-associated myopathy and improve neurologic scores in patients [80]. Although unclear, the primary mechanism of hCRF seems to be in the direct repair of BBB permeability and anti-angiogenic effects by agonism of CNS tumor CRF receptors, rather than its effect on systemic corticosteroids [84, 85]. These preclinical and early clinical findings have prompted multiple phase III clinical trials on Xercept (hCRF) *versus* dexamethasone for acute, chronic, and extended use cases, however, results have yet to be published NCT00226655, NCT00088166, NCT00226668. While the financial cost of hCRF treatment in this context has yet to be assessed, synthetic peptide therapies generally have a lower cost of production than antibodies such as bevacizumab, however, it is unlikely to approach the low cost of dexamethasone [86]. Additionally, both bevacizumab and hCRF require an IV or subcutaneous administration whereas steroid medications such as dexamethasone are available for oral administration in a shelf-stable form [87, 88].

### Boswellic acids

Boswellic acids (BAs) are a group of plant-derived compounds that have previously been studied in the context of the treatment of brain cancers and cerebral edema. Like many plant derivatives, the effects of BA are broad but include anti-inflammatory, antioxidant, and anti-tumor effects [89]. Variability in extraction methods, poor standardization, 91 makeup of extracts, and potential dietary interactions are barriers to the use of herbal medicine extracts [90, 91]. However, a patented *Boswellia serrata* extract given orally was shown to significantly reduce RT-related cerebral edema compared to a placebo with minimal drug-related adverse outcomes in a double-blind placebo-controlled trial [92]. In the trial, serum levels of measured BA’s varied greatly with some members of the experimental group having no measurable concentration of the presumed active extract product, 11-keto-β-BA [92]. However, concentrations of 11-keto-β-BA were highest among the patient with the highest measured reduction in edema [92]. Results found by another randomized control trial looking more broadly at serum levels of BA constituents suggested that β-BAs may be the primary active component of the extract as it was measured at steady serum levels [93]. The variability seen in these studies may result from the influence of participant diet, as the oral bioavailability of BA has been shown to increase when combined with dietary fat compared to when taken in a fasted state [94]. Despite the limitations of BA use discussed here, its low-cost, mild side effects, and oral route of administration make it an important potential steroid-sparing therapeutic for peritumoral and RT-related cerebral edema [90]. Future studies may reduce the pharmacokinetic variability using dietary instructions or through liposomal packaging [95].

Among trials comparing steroids to alternative agents for the treatment of cerebral edema, the heterogeneity in primary endpoints for treatment response is a major barrier to determining treatment efficacy [96]. Future study designs for corticosteroid alternatives must account for acute and long-term outcomes while keeping in mind the cost-to-benefit ratio of treatment.

## Pre-clinical investigation

Corticosteroids have been widely used to treat brain cancer by reducing cerebral edema perioperatively, targeting lymphomas in the CNS, and preventing or treating vomiting and nausea caused by chemotherapy [49]. The standard of care for treating gliomas, tumors that originate from glial cells, involves maximum surgical resection, external radiation, and chemotherapy [97]. Surgical resections result in brain injury and inflammation that breaks down the BBB and allows for the flow of fluid into the extracellular space of the parenchyma, causing extreme headaches and decreasing neurological function [23, 49], reducing the permeability of the BBB by upregulating tight junction components in endothelial cells (see Figure 1), thus decreasing the intracranial pressure [49, 98]. Steroid treatment of CNS lymphomas has been shown to be effective in inducing tumor cell apoptosis [49]. As a result of chemotherapy, many patients experience nausea and vomiting that can dehydrate and decrease electrolyte levels which, if left untreated, can lead to death. Steroids, specifically dexamethasone, reduce the release of serotonin 5-hydroxytryptamine3 (5-HT_3_) from the gastrointestinal (GI)-tract and contributes to these symptoms [49]. Treatment for brain cancer doesn’t solely rely on steroids, rather the glucocorticoid activity must work concurrently with radiation, chemotherapy, and immunotherapy to efficiently treat cancer. Studies have shown that long-term use of steroids leads to immune system impairment and steroid-induced diabetes that reduces immunotherapy efficiency and increases glucose availability for tumor cell metabolism [99, 100]. Due to these effects, studies should be focused on identifying novel treatments that reduce cerebral edema and inflammation without inhibiting the efficiency of a treatment administered concurrently.

**Figure 2.**
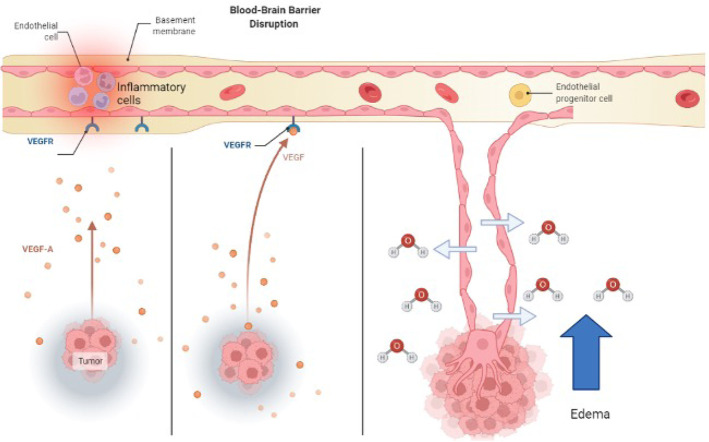
How angiogenic signaling molecules cause new faulty vessels to develop. New vessels are highly permeable to fluid and extravasation contributes to vasogenic edema. VEGFR: VEGF receptor; VEGF-A: VEGF factor A

Glioblastoma, a common and extremely aggressive brain tumor among adults, is continuously being studied for more targeted therapy to increase the survival rate of patients [100]. Immunotherapy with checkpoint inhibitors has seen promising results in treating cancer by specifically targeting CTLA-4 and PD-L1 on T cells, upregulating T-cell antitumor activity [57]. After surgical resection, the BBB becomes disrupted with an increase of pro-inflammatory cytokine and chemokine release, as well as monocyte trafficking and infiltration to the inflammatory site (see Figure 2) [101]. Liu et al. [31] demonstrated that blocking the receptor for advanced glycation end products (RAGE), a multiligand receptor associated with prolonged inflammation, and upstream inflammatory proteins like S100B and high motility group box 1 (HMGB1) prevented the breakdown of the BBB by suppressing neuroinflammation. It is important to note that Liu et al.’s [31] study also showed that the RAGE inhibitor didn’t affect the efficiency of the anti-PD-1 checkpoint inhibitor, but dexamethasone did reduce the efficiency of this therapy. Intratumoral viral oncolytic immunotherapy has also been investigated in the treatment of brain tumors. This type of therapy involves genetically modifying oncolytic viruses to reduce tumor pathogenicity [98, 102] showed that a high dosage of dexamethasone also reduced the potency of these oncolytic adenoviruses by increasing neutrophils and the proportion of tumor-infiltrating macrophages.

**Figure 3.**
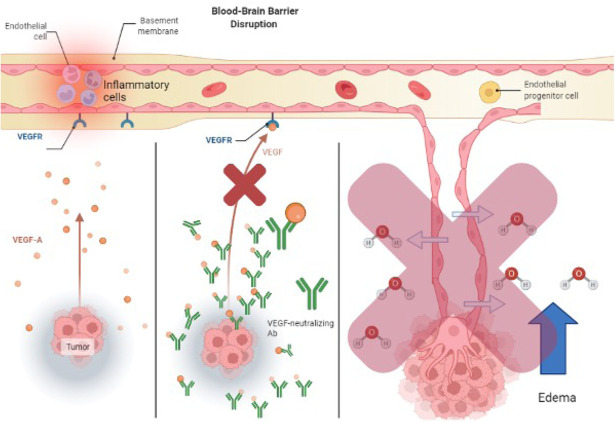
How VEGF neutralizing antibodies act to mediate vasogenic edema. This is distinctly different than steroids increasing tight junction expression

Two other alternatives to steroid treatment are VEGF-neutralizing antibodies and corticorelin acetate. As tumor cells proliferate and develop, VEGF-A becomes upregulated and deformed blood vessels form for oxygen and nutrients [103]. These new blood vessels don’t have the barrier integrity that tight junctions have and allow for the transport of water and molecules into the brain (see Figure 2). Bevacizumab is an immunoglobulin G (IgG) monoclonal antibody that binds to VEGF-A and inhibits its endothelial cell proliferation (see Figure 3) with no effect on chemotherapy efficiency [97]. Another treatment for brain tumors involves the development of peptides used directly as bioactive therapeutics because of their facilitated access through the cell and tissue barriers, thus improving the delivery of drugs past the BBB [80, 104]. An early clinical trial has shown that the administration of hCRF reduced peritumoral edema independent of steroid use [104]. Corticorelin acetate is a synthetic peptide formed on normal endogenous hCRF that has been shown to decrease vascular leakage and increase the integrity of the BBB [79]. The exact mechanism for this peptide is still unknown, however, its functionality might revolve around anti-angiogenic mechanisms that inhibit tumors from forming deformed blood vessels [79].

## Conclusions

Steroids remain a powerful tool to provide rapid symptomatic relief from tumor-associated edema and radiation necrosis. Careful patient selection and judicious use of steroids are recommended to optimize benefits while reducing side effects. Alternative agents such as bevacizumab, corticorelin, and BAs have been used but various barriers remain to further implementation. There is a paucity of data regarding ICIs and concurrent steroid administration. Further research is needed to develop an algorithm for steroid cessation to optimize therapeutic efficacy and improve patient outcomes.

## Abbreviations

BAs: boswellic acids
BBB: blood-brain barrier
CNS: central nervous system
CRF: corticotropin-releasing factor
GBM: glioblastoma multiforme
hCRF: human corticotropin-releasing factor
ICIs: immune checkpoint inhibitors
MP: methylprednisolone
NCCN: National Comprehensive Cancer Network
NSCLC: non-small cell lung cancer
PD-1: programmed cell death protein 1
RBN: radiation-induced brain necrosis
RT: radiotherapy
TMZ: temozolomide
VEGF: vascular endothelial growth factor
VEGF-A: vascular endothelial growth factor factor A

## Declarations

### Copyright

© The Author(s) 2022.
